# Supplementary material for: Single-cell analysis reveals host S phase drives large T antigen expression during BK polyomavirus infection
Source: PLoS Pathog. 2024 Dec 5;20(12):e1012663. doi: 10.1371/journal.ppat.1012663 (PMC11620372; doi:10.1371/journal.ppat.1012663)
Supplement: S1 Fig — (DOCX) [file ppat.1012663.s001.docx]

**
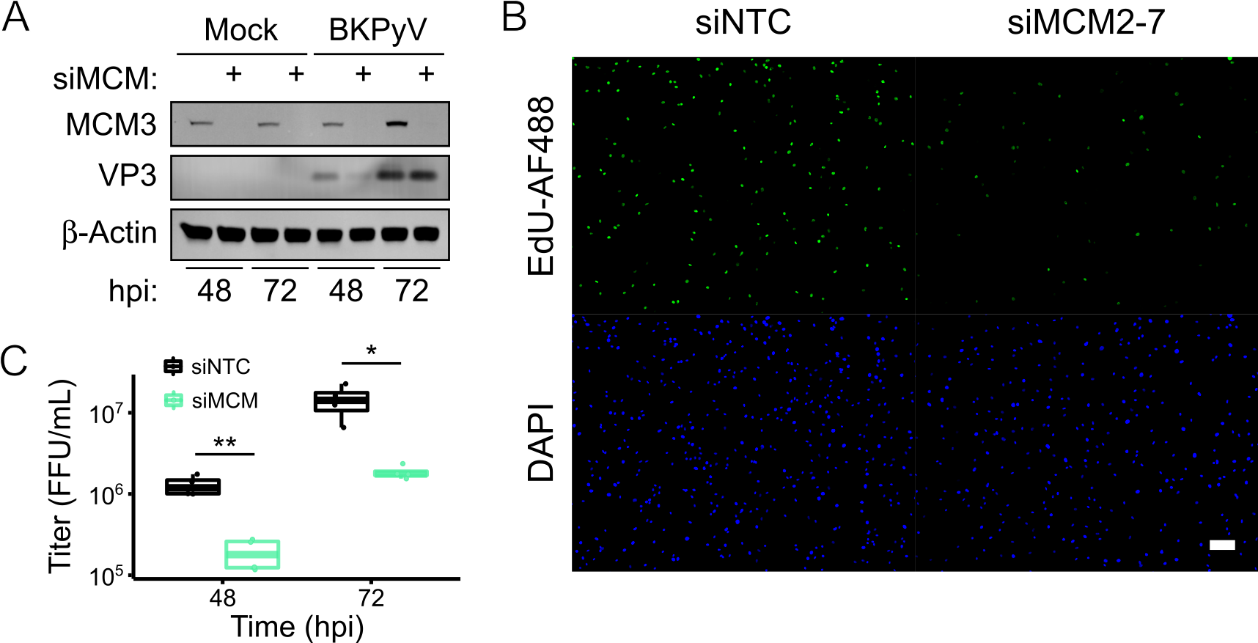
**

**S1 Fig. MCM knockdown decreases BKPyV production.** (A) Representative western analysis (n=3) of RPTE cells transfected with either siRNA against MCM2-7 (siMCM) or a non-targeting control (siNTC) siRNA 24 hours prior to infection with BKPyV (MOI=0.5) or mock-infected. (B) Representative IFA image (n=3) of BKPyV infected RPTE cells transfected with a non-targeting control (siNTC) or siRNA targeting MCM2-7. Cells were infected at 24 hours after siRNA transfection and were pulse-labeled with EdU for 3 hours and fixed at 48 hours post-infection. EdU was visualized by click-it reaction with AF488 azide. Scale bar represents 100μm. (C) Viral titers from virus collected at 48 or 72hpi from siNTC or siMCM RPTE cells (n=3). Statistical significance was determined by multi-factorial ANOVA and a Tukey post-hoc (* : p < 0.05, ** : p < 0.01).
