# Supplementary material for: Single-cell analysis reveals host S phase drives large T antigen expression during BK polyomavirus infection
Source: PLoS Pathog. 2024 Dec 5;20(12):e1012663. doi: 10.1371/journal.ppat.1012663 (PMC11620372; doi:10.1371/journal.ppat.1012663)
Supplement: S2 Fig — (DOCX) [file ppat.1012663.s002.docx]

**
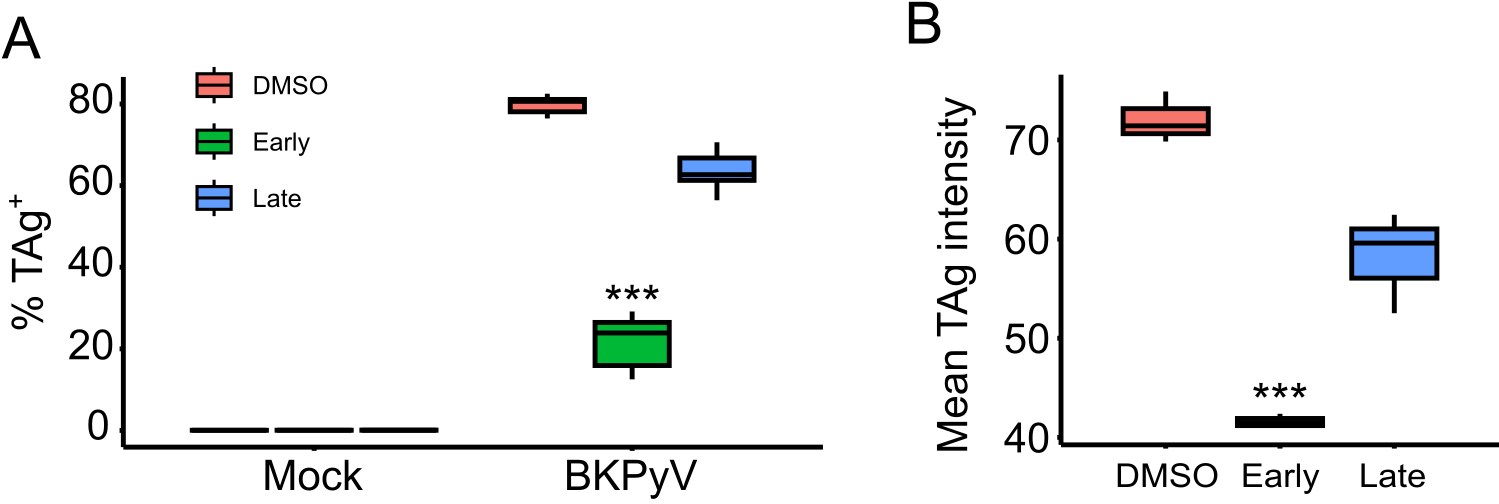
**

**S2 Fig. MCM inhibition early, but not late, decreases TAg expression.** RPTE cells were treated with the MCM inhibitor heliquinomycin (3μM) either early (18hpi) or late (48hpi) and cells were fixed at 72hpi (n=3). (A) Quantification of percent TAg+ in mock or BKPyV infected cells. (B) TAg intensity of BKPyV-infected, TAg+ cells from panel A. Statistical significance was determined by one-way ANOVA and a Tukey post-hoc (*** : p < 0.001).
