# Supplementary material for: Single-cell analysis reveals host S phase drives large T antigen expression during BK polyomavirus infection
Source: PLoS Pathog. 2024 Dec 5;20(12):e1012663. doi: 10.1371/journal.ppat.1012663 (PMC11620372; doi:10.1371/journal.ppat.1012663)
Supplement: S3 Fig — (DOCX) [file ppat.1012663.s003.docx]

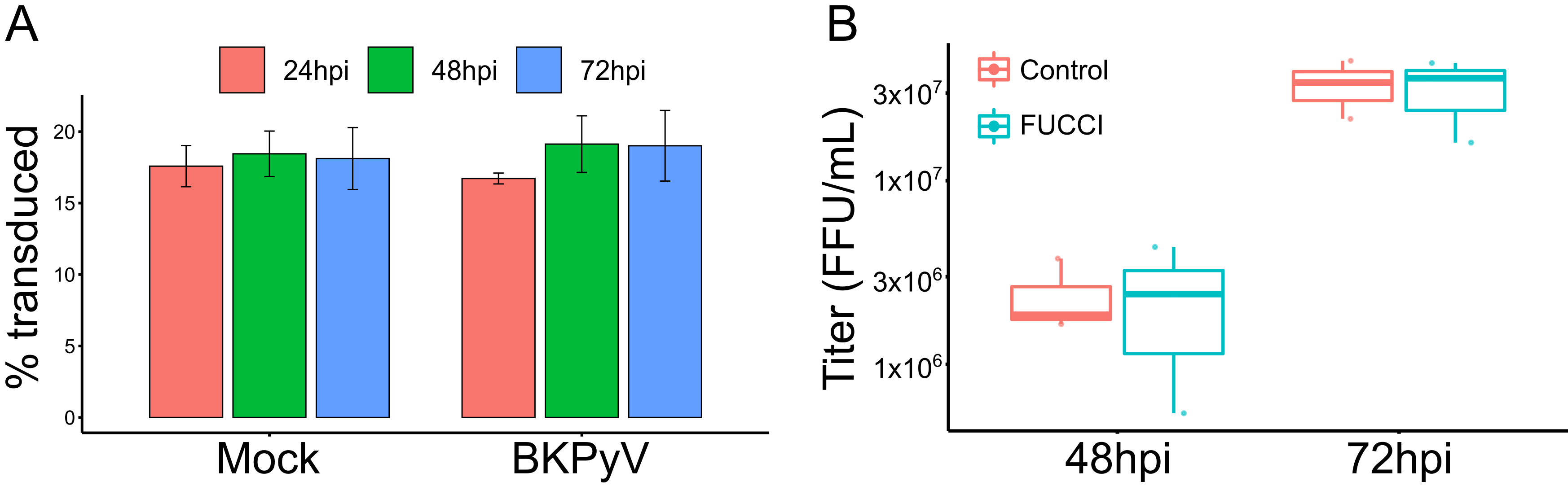


**S3 Fig. FastFUCCI transduced RPTE cells do not impair BKPyV infection.** (A) Quantification of the percent of RPTE cells expressing either a red or a green signal above background in mock or BKPyV-infected RPTE cells at 24, 48, and 72hpi (n=3). (B) Quantification of viral titers collected from either mock or FastFUCCI transduced RPTE cells at 48 and 72 hpi. Titers were quantified by focus forming assay (n=3).
