# Supplementary material for: Single-cell analysis reveals host S phase drives large T antigen expression during BK polyomavirus infection
Source: PLoS Pathog. 2024 Dec 5;20(12):e1012663. doi: 10.1371/journal.ppat.1012663 (PMC11620372; doi:10.1371/journal.ppat.1012663)
Supplement: S4 Fig — (DOCX) [file ppat.1012663.s004.docx]

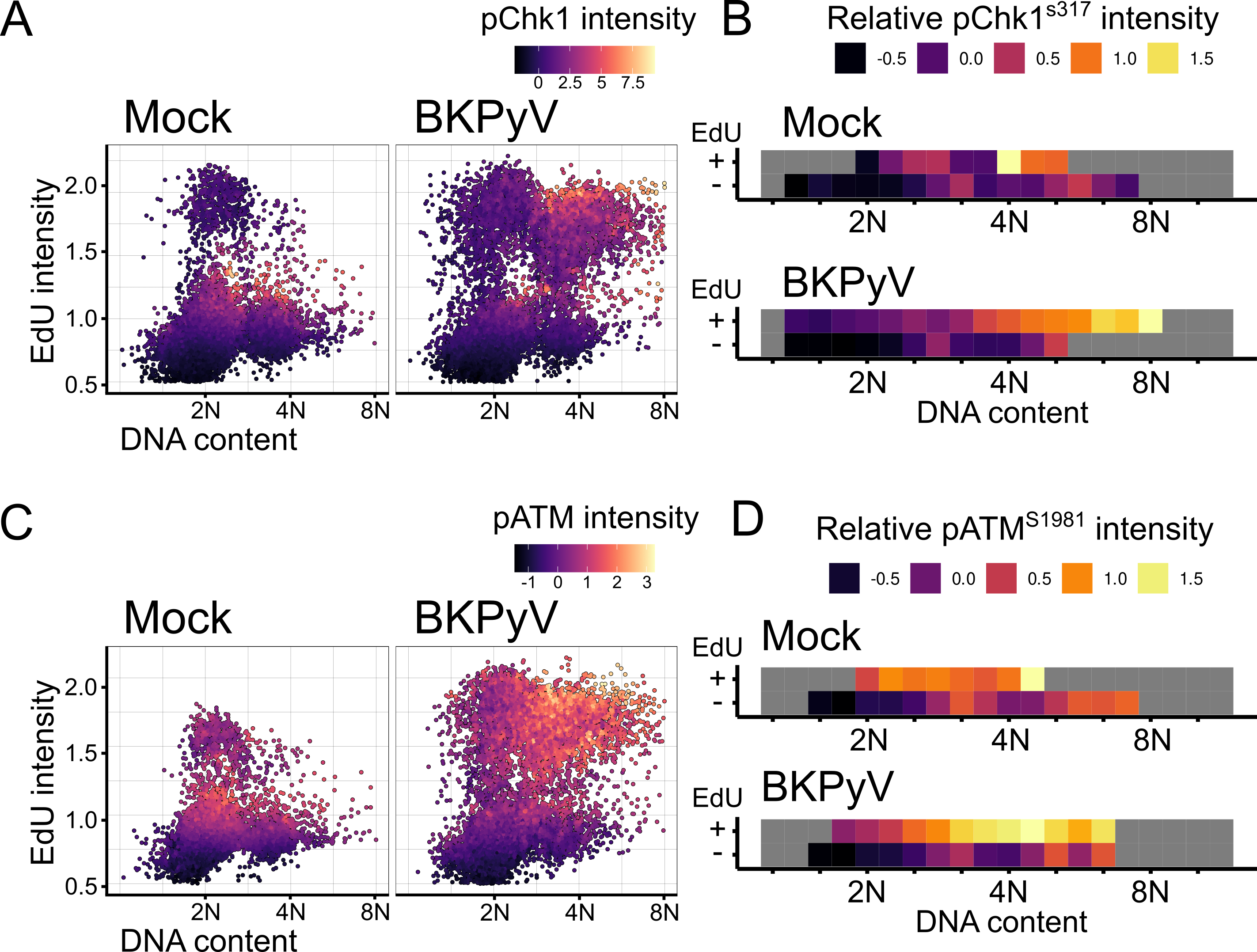


**S4 Fig. ATR and ATM activation primarily found in re-replicating cells.** (A) Representative (n=3) cell cycle plot of mock or BKPyV-infected RPTE cells at 48hpi were colored based on the mean nuclear intensity of the ATR marker pChk1^S317^. (B) Representative binned pChk1 intensity plot of data in panel A. Bin color was determined by averaging pChk1 nuclear intensity of all cells within the bin. (C) Representative cell cycle plot of mock or BKPyV-infected RPTE cells at 48hpi and colored based on nuclear intensity of the ATM marker pATM^S1981^. (D) Representative binned pATM intensity plot of data in (C). Bin color was determined by averaging pATM nuclear intensity of all cells within the bin.
