# Supplementary material for: Single-cell analysis reveals host S phase drives large T antigen expression during BK polyomavirus infection
Source: PLoS Pathog. 2024 Dec 5;20(12):e1012663. doi: 10.1371/journal.ppat.1012663 (PMC11620372; doi:10.1371/journal.ppat.1012663)
Supplement: S5 Fig — (DOCX) [file ppat.1012663.s005.docx]

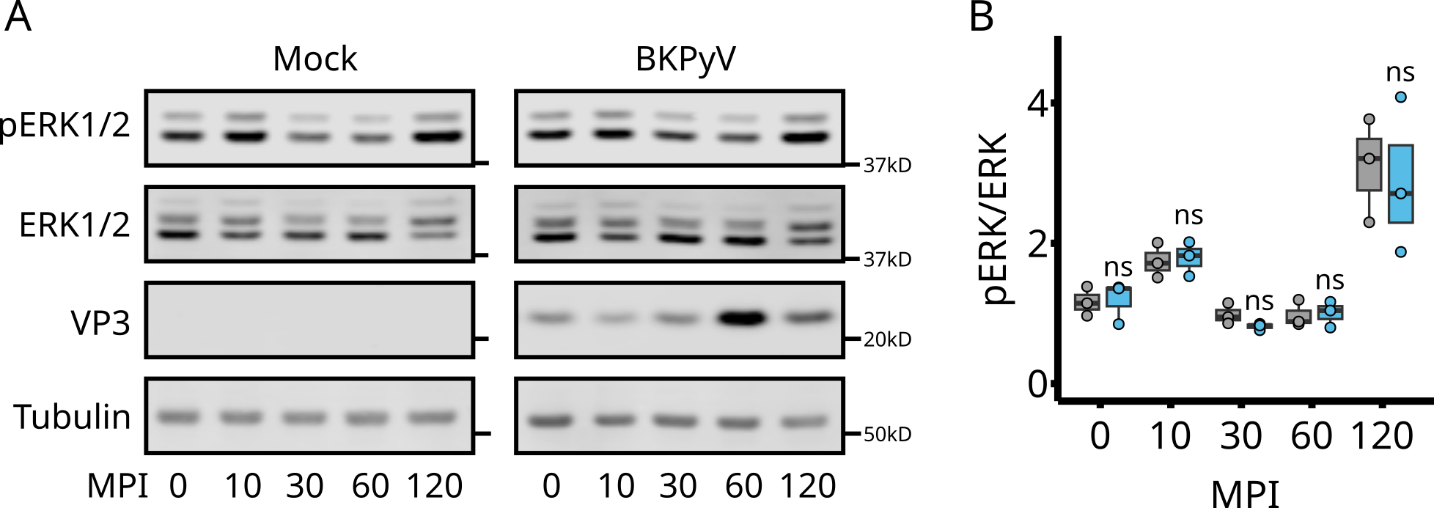


**S5 Fig. BKPyV binding during infection does not activate MAPK pathway.** (A) RPTE cells were infected with BKPyV (MOI=0.5) for 1 hour at room temperature. Immediately following infection, the cells were washed, returned to fresh media, and incubated at 37^o^C/5% CO_2_. At the indicated time points (mpi: minutes post-infection), cells were collected for western analysis probing for the MAPK Erk1/2, the active phosphor-form (pERK1/2), the loading control tubulin, and the viral capsid protein VP3. Western analysis is representative of n=3. (B) Quantification of the ratio of active Erk1/2 to total Erk1/2 represented in (A). Significance was tested by Student’s t-test comparing the mock-infected samples to the BKPyV-infected samples at each timepoint.
