# Supplementary material for: Single-cell analysis reveals host S phase drives large T antigen expression during BK polyomavirus infection
Source: PLoS Pathog. 2024 Dec 5;20(12):e1012663. doi: 10.1371/journal.ppat.1012663 (PMC11620372; doi:10.1371/journal.ppat.1012663)
Supplement: Attachment — Submitted filename: ReponseToReviewersv3.docx [file ppat.1012663.s006.docx]

We would like to thank the reviewers for their careful reading and constructive comments to improve our manuscript. In particular, we would like to thank them for finding our data convincing, the manuscript was well-written and that the data is rigorous and clearly presented. We have addressed all of their comments and feel that the manuscript is greatly improved.

**Reviewer 1**

*The manuscript by Needham et al describes studies on the initial replication of BK virus (BKPyV) after infection of primary human kidney cells (RPTE). The major conclusion is that the viral large T-antigen (LTag) stimulation of host cell replication follows an initial host cell S-phase, which is unrelated to viral protein expression. Then, once the viral proteins are expressed following this initial S-phase induction, viral replication becomes directed by LTag and is independent of host cell replication. The data is convincing with respect to the expression of LTag for re-replication. However, as observed in infection by other polyomaviruses (e.g., SV40, MuPyV) virion binding to cells alone initiates a mitogenic response, inducing multiple serum response genes (e.g, c-myc, c-fos, etc.). Thus, although LTag is responsible for subsequent cell cycle disruption the virus appears to rely upon virion attachment to initiate infection. In a sense therefore the conclusions here are correct, but the initial activating source of cell cycle induction, which is the virus itself, is a missing part of the experiments and discussion. The mock controls shown are insufficient to address this important point, and a necessary control would be BKV VLPs (no genomes) to demonstrate the independent mitogenic activity of virus attachment.*

**The reviewer correctly points out that virion binding has been demonstrated to activate mitogenic responses in other polyomaviruses. This activation of mitogen activated kinases (MAPKs) could cause an increase in S phase, which we demonstrate in this study is required for TAg expression and subsequent BKPyV replication. Furthermore, BKPyV has been shown to require ERK activity early during infection. To test if BKPyV similarly activated the MAPK pathway following infection, we looked for MAPK activation immediately following our infection protocol but found no difference in mitogenic signaling between uninfected and BKPyV infected cells. We have included this as a new Supplemental Figure 5, and include the following text in the discussion (lines 621-628): “**Finally, it may be that virus entry is sufficient to promote a burst of S phase induction. Indeed, MAPK inhibition during infection has been shown to decrease polyomavirus infection. Furthermore, SV40 and JCPyV attachment alone is sufficient to promote MAPK activation. While we did not find that BKPyV infection increased MAPK activation following attachment (Figure S5) nor did we observe any difference in S phase induction until >24 hours post-infection (Figure 1), it is possible that a later step during infection, such as escape from the endoplasmic reticulum or nuclear entry, activates MAPK to drive the initial S phase prior to BKPyV transcription.**”. If we had observed an increase in MAPK activation after BKPyV infection, we planned to perform the experiment suggested by the reviewer using empty virions to determine if the initial S phase was triggered by attachment. However, since we do not observe an increase in MAPK activation, we feel this experiment is unnecessary for this study. Furthermore, there is no discernible difference in S phase induction between uninfected and BKPyV infected cells until the 36hpi time point. If attachment indeed triggered mitogenic signaling to drive S phase induction, we would expect an earlier, more gradual increase in S phase. Instead, as it shown in Figure 1B-C, the initial S phase occurs synchronously more than a day after infection. The reviewer similarly notes that the “data is convincing with respect to the expression of LTag”, which is the main claim of this study. Understanding the mechanism driving this initial S phase induction remains a goal of our lab and we are conducting experiments to determine the contribution of the infection environment on viral replication in a cell-cycle dependent manner. However, elucidating that mechanism within this manuscript is both beyond the scope of this work and would only further increase the size of this manuscript.**

**Reviewer 2**

*This interesting study uses single cell analysis to examine the effect of cell cycle on large T antigen expression during BK polyomavirus infection. The accepted model in the field is that TAg is expressed early to promote cell cycle transition into S phase. Here they suggest that TAg expression requires host DNA replication. They propose a new model in which TAg expression depends upon an initial host S phase, and that the viral genome is replicated primarily during a subsequent phase of host re-replication. The manuscript is well written, the data are rigorous and clearly presented, and the interpretations are thoughtful and mostly supported by the data. They use a powerful combination of elegant approaches. There are some caveats, but these are thoughtfully considered. The results are likely to stimulate new directions of research in this area.*
**We want to thank reviewer 2 for their kind words and careful reading of the manuscript. We have fully addressed their comments below and believe that the manuscript is greatly improved.**

*Figure 1 examines where TAg is expressed in cell cycle by EdU labeling and single cell cycle plots combined with immunofluorescence. TAg accumulates in the re-replicating cells. Could it be that TAg made in newly infected cells is less stable that TAg made in S-phase and this explains the difference in intensity and low amount at early times? This is partly addressed by later experiments but differential stability is not fully discussed.*

**The reviewer rightly points out the possibility that TAg stability may be affected by the host cell cycle state. The purpose of figure 1 is to establish the spatial pattern of TAg expression, while potential mechanisms are explored later. Indeed, as we reference in the lines 363-364, Merkel polyomavirus TAg has been shown to be destabilized by the MCV LT unique region. For this reason, we performed proteasome inhibition of BKPyV infected primary cells in Figure 3H-I, which failed to increase TAg abundance by western analysis. If BKPyV TAg was unstable, we would have observed an increase in protein abundance. To reinforce this point, the following lines have been added to the discussion (lines 585-587): “**Proteasome inhibition failed to increase TAg expression suggesting that the increase of BKPyV TAg in G2/M is not due to a shift in protein stability, as has been reported for the related MCPyV^36^.**”**

*In Figure 2 they use inhibitors to block the initial S phase and compare to inhibition at late times. These data suggest BKPyV requires the initial host S phase for virus production. Do they have controls to demonstrate that the inhibitors have been effective? The pulse labeling experiment in Fig 2D is particularly elegant.*

**The reviewer is correct to suggest that we show our mock S phase levels under inhibitor treatment (Cdc7, Cdk2, and MCM inhibition). The S phase levels for both uninfected and BKPyV infected cells under Cdc7 or Cd2 inhibition are shown in Figure 5G-I. While we considered moving these quantifications up to Figure 2, we feel that S phase levels are better placed in Figure 5 to demonstrate the necessity of these kinases for S phase initiation. However, since we did not include the effect of MCM inhibition on S phase levels for uninfected or BKPyV infected cells, these quantifications have been added to Supplemental Figure 2. We have also added representative immunofluorescent microscopy images of the MCM2-7 siRNA knockdown cells to Supplemental Figure 1, which show a subsequent loss in S phase.**

*Have they done anything to analyze viral gene expression at the transcription level (e.g. FISH) during these different cell cycle stages?*

**We have not analyzed viral gene transcription across cell cycle stages, but we agree that this would be an excellent experiment to determine if the cell cycle-dependent expression of TAg was due to changes in transcription. While we plan to perform these experiments in a future project to identify the exact mechanism of robust TAg expression, this study was focused on demonstrating that BKPyV TAg expression is cell-cycle dependent.**  *Figure 3 uses increasing MOIs to account for viral genome copy number. Did they check that higher MOIs correlates with the expected amount of viral genomes uncoated in the nucleus?*

**The reviewer rightly points out that we did not confirm the increase in MOI with uncoated genomes in the nucleus in Figure 3. Since one of the conclusions of Figure 3 is that *the distribution of TAg* throughout the cell cycle is insensitive to genome copy number, it is possible that a linear increase in MOI (as calculated by the number of cells that are TAg+ by 48hpi) does not correlate with nuclear translocation of BKPyV genomes. For example, there may be a physiological limit on the number of virions capable of escaping the ER such that there exists an MOI at which the nucleus is “saturated”. However, we note that both the overall S phase levels and TAg positivity increase with MOI from MOI=0.01 to MOI=10 suggesting that a “saturated” state has not been reached. Furthermore, the observation that the cell cycle distribution of TAg+ cells does not substantially change over this range supports the conclusion that the increase in TAg is not a simple function of genome copy number.**

*Have they considered that there could be a repressor preventing TAg gene expression in the TAg-ve cells?*

**We had not considered the possibility of a TAg repressor although it is an excellent suggestion for future work. While the binding of TAg to pRb-family proteins is often considered in the context of removing the repression of E2F transcriptional activators, the pRb-related protein p130 is known to act in the DREAM complex for long term transcriptional repression. This may provide a mechanism for a proposed transcriptional activation of TAg, as the transition out of quiescence requires disassembly of the DREAM complex. However, Cdc7 and MCM inhibition, as well as MCM knockdown, prevented TAg accumulation. As we are unaware of evidence that neither Cdc7 nor the MCM helicase are required for DREAM disassembly, this is an unlikely mechanism. Another major class of cell-cycle transcriptional repression are the atypical E2F repressors E2F7/8, which are expressed in S phase to repress the activating E2Fs. However, since neither E2F7 nor E2F8 have been found to be expressed outside of S phase, this too seems an unlikely mechanism for repressing TAg transcription.**

*Have they also considered labeling nascent proteins and immunoprecipitating for TAg to see whether alterations in protein synthesis levels could be a contributing factor?*

**We have used nascent protein labeling using both ­^35^S-methionine/cysteine pulse labeling (manuscript submitted) and an alkyne-containing methionine analog (manuscript in preparation) that show little changes in global translation throughout a BKPyV infection. Still, it is possible that TAg translation itself is specifically blocked prior to late S/G2 phase. We plan on exploring this potential mechanism in our future work that will build on these findings.** *Figure 4 looks at the requirements of origin licensing factors and Figure 5 examines firing of new origins. These are elegant but somewhat complex experiments. The interpretations are cautious and suggest that canonical mechanisms are used for origin licensing and firing.*

**We thank the reviewer for their kind words about our work.** *Figure 6 and the Discussion present their new model which requires S phase for TAg expression. Here they discuss alternative splice variants and truncated Tags. They could speculate a little more about what activates TAg expression in S/G2, e.g. specific transcription factors, altered translational control, loss of a repressor etc.*

**We have added the following text to the discussion to further speculate on the specific activation of TAg (lines 631-648):** “While we have shown here that the cell cycle-specific accumulation of TAg is independent of proteasomal degradation and is insensitive to viral genome numbers, the mechanism underlying this rapid increase in TAg remains unclear. If TAg splicing is cell cycle dependent, it may be that the other splice variants, truncTAg and tAg, predominate until G2/M when TAg is preferentially spliced. Alternatively, BKPyV early gene transcription itself may depend on host transcription factors that are preferentially activated during late S phase, such as the MuvB/B-Myb (MMB) complex. Interestingly, MuvB is also a component of the DREAM complex, which complexes with the pRb paralogs 107/p130 to repress cell cycle transcription. TAg binding to the pRb family members may then serve multiple functions through increasing cell cycle entry by dismantling the DREAM complex, promoting S phase entry by binding pRb, and increasing G2/M transcription by promoting MMB formation. If the BKPyV early promoter recruited MMB complexes, this could result in a feedback loop to rapidly increase transcription of the TAg mRNA in late S/G2. Finally, there may exist cell cycle-specific changes in TAg translation that decouple TAg mRNA abundance from protein abundance. For example, noncanonical translation initiation has been reported to be altered across the cell cycle. Alternatively, TAg mRNA stability may be cell cycle dependent through an unknown mechanism.”

Minor comments:

Reviewer #1: Other Comments:
1. For BKV “reactivation”, it would be helpful to clarify (at least comment on, since likely unknown) where the virus is being reactivated from, and how the virus may be maintained in cells before reactivation. Although briefly addressed in the final sentences of the conclusions (lines 626-633), this biology seems important to the possible in vivo relevance of these experiments. **The author is correct in surmising that the mechanisms and specific cellular tropisms behind BKPyV reactivation are unknown. It is known that BKPyV persistently infects the proximal tubule epithelial cells within the kidney, which is stated in the introduction. To make this even more clear, we have added the “**the kidneys of**” (line 73) to the opening line of the introduction, and added the following sentence to the discussion to better point out the current unknowns surrounding BKPyV infections in vivo (lines 689-690): “**Similarly, the mechanisms regulating BKPyV reactivation are unknown, as is much of the viral lifecycle in vivo.”**.**

2. “Robust levels” of LTag are not explained more than immunofluorescent measurements. Another more quantitative assay for LTag would be useful. At the least, estimate the level of detection. **We recognize that there are limits to our conclusions since we do not know how much TAg expression is required to detect this protein by IFA. However, we addressed this in two ways. First, we qualified the TAg expression as “robust” since we are unable to rule out low expression earlier and we even stated this. Second, we inhibited the first S phase, which should show gradual increases in TAg expression if it was just low levels that needed time to build up, but this is not what we observed. Rather even after 54 hours we did not observe any accumulation in TAg expression in cells that were in G1/G0 (Figure 2F). So, while we were unable to figure out how to quantify the levels of TAg expressed in a single-cell we accepted the caveats of our data and tempered our conclusions. Unfortunately, the TAg antibody does not work in FACS so we are unable to sort cells based on TAg expression levels.**

3. The figures are extremely complex (5 x 9 panels each!)! Although the data are appreciated, the presentation might/should be clarified. Perhaps move some related panels to the appendix, or combine related panels into unified figures? **Granted we agree that this manuscript is complicated and has a lot of data, but we carefully evaluated the panels for each figure and did our best to make them easy to follow and to step the reader through the data. We understand that the manuscript is complicated but removing panels would leave holes and may lose the reader. We even considered breaking the manuscript into 2 separate manuscripts, but this would not allow us to present a cohesive story to demonstrate that S phase drives large TAg expression.**

*3. A stylist comment: “this” without a subject is often confusing. Please correct.* **We have corrected this by using more descriptive words. We appreciated the suggestion and have removed >50% of them.**

4. References to similar experiments with other polyomaviruses would be helpful. **We reference various other polyomavirus-related publications and experiments throughout the work. To our knowledge, this work represents the first clear evidence of cell-cycle regulated expression of TAg. For BKPyV specifically, we cite a previous publication that found heterogenous expression of TAg using single cell transcriptomics (reference 26), while another group found a correlation between larger nuclei and TAg expression (reference 27). For other PyVs, we cite experiments involving SV40, JCPyV, and MCPyV.**

*Reviewer #2: Line 273 is missing a word?* **This is fixed, thank you.**
